# Supplementary material for: Near-infrared dual bioluminescence imaging in mouse models of cancer using infraluciferin
Source: eLife. 2019 Oct 15;8:e45801. doi: 10.7554/eLife.45801 (PMC6828332; doi:10.7554/eLife.45801)
Supplement: Transparent reporting form [file elife-45801-transrepform.pdf]

1st Floor
24 Hills Road
Cambridge CB2 1JP, UK

P 01223 855340
W elifesciences.org
T @elife

eLife's transparent reporting form

We encourage authors to provide detailed information *within their submission* to facilitate the interpretation and replication of experiments. Authors can upload supporting documentation to indicate the use of appropriate reporting guidelines for health-related research (see EQUATOR Network), life science research (see the BioSharing Information Resource), or the ARRIVE guidelines for reporting work involving animal research. Where applicable, authors should refer to any relevant reporting standards documents in this form.

If you have any questions, please consult our Journal Policies and/or contact us: editorial@elifesciences.org.

Sample-size estimation

- You should state whether an appropriate sample size was computed when the study was being designed
- You should state the statistical method of sample size computation and any required assumptions
- If no explicit power analysis was used, you should describe how you decided what sample (replicate) size (number) to use

Please outline where this information can be found within the submission (e.g., sections or figure legends), or explain why this information doesn't apply to your submission:

Details on sample size decision can be found in materials and methods (sections *In vitro* bioluminescence assays and *In vivo* models).
In applicable experiments, sample size can be found in the figure legends (figure 2, 4, 5 and 6).

Replicates

- You should report how often each experiment was performed
- You should include a definition of biological versus technical replication
- The data obtained should be provided and sufficient information should be provided to indicate the number of independent biological and/or technical replicates
- If you encountered any outliers, you should describe how these were handled
- Criteria for exclusion/inclusion of data should be clearly stated
- High-throughput sequence data should be uploaded before submission, with a private link for reviewers provided (these are available from both GEO and ArrayExpress)

Please outline where this information can be found within the submission (e.g., sections or figure legends), or explain why this information doesn't apply to your submission:

In applicable experiments, information on replicates can be found in materials and methods (section *In vitro* bioluminescence assays and figure legends (figure 2)).
The synthetic chemistry was performed as written in the Supplementary Information. Each step will have been repeated 3-5 times and optimized to some extent during these. The yields quoted are of the average experiment, that is not the lowest or highest yields, but the one most often obtained.

eLife Sciences Publications, Ltd is a limited liability non-profit non-stock corporation incorporated in the State of Delaware, USA, with company number 5030732, and is registered in the UK with company number FC030576 and branch number BR015634 at the address 1st Floor, 24 Hills Road, Cambridge CB2 1JP | August 2014

Statistical reporting

- Statistical analysis methods should be described and justified
- Raw data should be presented in figures whenever informative to do so (typically when N per group is less than 10)
- For each experiment, you should identify the statistical tests used, exact values of N, definitions of center, methods of multiple test correction, and dispersion and precision measures (e.g., mean, median, SD, SEM, confidence intervals; and, for the major substantive results, a measure of effect size (e.g., Pearson's r, Cohen's d)
- Report exact p-values wherever possible alongside the summary statistics and 95% confidence intervals. These should be reported for all key questions and not only when the p-value is less than 0.05.

Please outline where this information can be found within the submission (e.g., sections or figure legends), or explain why this information doesn't apply to your submission:

Statistical methods are outlined in the materials and methods section 'Statistical analysis'. In addition, details of specific statistical methods and values can be found in sections 'Spectral unmixing of firefly luciferase mutants *in vitro*', 'Spectral characterisation of firefly luciferase mutants with LH_2 and iLH_2 *in vivo*', 'Spectral unmixing of firefly luciferase mutants *in vivo*' and the legend of Figure 4.

(For large datasets, or papers with a very large number of statistical tests, you may upload a single table file with tests, Ns, etc., with reference to sections in the manuscript.)

Group allocation

- Indicate how samples were allocated into experimental groups (in the case of clinical studies, please specify allocation to treatment method); if randomization was used, please also state if restricted randomization was applied
- Indicate if masking was used during group allocation, data collection and/or data analysis

Please outline where this information can be found within the submission (e.g., sections or figure legends), or explain why this information doesn't apply to your submission:

For both *in vitro* and *in vivo* experiments the same cells/mice were tested with both LH_2 and iLH_2, so group allocation does not apply to most of this submission. The only exception is the experiment carried out in the section 'Application of dual bioluminescence imaging using infraluciferin' where details of group allocation can be found in the legend of figure 6.

Additional data files ("source data")

- We encourage you to upload relevant additional data files, such as numerical data that are represented as a graph in a figure, or as a summary table
- Where provided, these should be in the most useful format, and they can be uploaded as "Source data" files linked to a main figure or table
- Include model definition files including the full list of parameters used
- Include code used for data analysis (e.g., R, MatLab)
- Avoid stating that data files are "available upon request"

eLife Sciences Publications, Ltd is a limited liability non-profit non-stock corporation incorporated in the State of Delaware, USA, with company number 5030732, and is registered in the UK with company number FC030576 and branch number BR015634 at the address 1st Floor, 24 Hills Road, Cambridge CB2 1JP | August 2014

Please indicate the figures or tables for which source data files have been provided:

Due to the specialized nature of the raw images generated in this research, and the software needed to analyse these images, raw image files for figures 2, 4, 5 and 6 can be made available upon request.
